# Molecular characterization of the whole genome of H9N2 avian influenza virus isolated from Egyptian poultry farms

**DOI:** 10.1007/s00705-024-06018-2

**Published:** 2024-04-16

**Authors:** Nahed M. Bedair, Moustafa A. Sakr, Ahmed Mourad, Nourhan Eissa, Ahmed Mostafa, Omaima Khamiss

**Affiliations:** 1 Molecular Diagnostics and Therapeutics Department, Genetic Engineering and Biotechnology Research Institute (GEBRI), University of Sadat City (USC), Sadat, Egypt; 2 Department of Surgery, Anesthesiology and Radiology, Faculty of Veterinary Medicine, University of Sadat City, Sadat, Egypt; 3 Department of Animal Hygiene and Zoonoses, Faculty of Veterinary Medicine, University of Sadat City, Sadat, Egypt; 4 Center of Scientific Excellence for Influenza Viruses, National Research Centre, 12622 Dokki, Giza Egypt; 5 Animal Biotechnology Department, Genetic Engineering and Biotechnology Research Institute (GEBRI), University of Sadat City (USC), Sadat, Egypt

**Keywords:** H9N2, Phylogenetic analysis, Virulence, Mammalian preference

## Abstract

H9N2 avian influenza viruses (AIVs) affect both poultry and humans on a global level, and they are especially prevalent in Egypt. In this study, we sequenced the entire genome of AIV H9N2 isolated from chickens in Egypt in 2021, using next-generation sequencing (NGS) technology. Phylogenetic analysis of the resulting sequences showed that the studied strain was generally monophyletic and grouped within the G1 sublineage of the Eurasian lineage. Four segments (polymerase basic 2 [PB2], polymerase basic 1 [PB1], polymerase acidic [PA], and non-structural [NS]) were related to Egyptian genotype II, while the nucleoprotein (NP), neuraminidase (NA), matrix (M), and haemagglutinin (HA) segments were related to Egyptian genotype I. Molecular analysis revealed that HA protein contained amino acid residues (191H and 234L) that suggested a predilection for attaching to human-like receptors. The antigenic sites of HA had two nonsynonymous mutations: V194I at antigenic site A and M40K at antigenic site B. Furthermore, the R403W and S372A mutations, which have been observed in H3N2 and H2N2 strains that caused human pandemics, were found in the NA protein of the detected strain. The internal proteins contained virulence markers: 504V in the PB2 protein, 622G, 436Y, 207K, and 677T in the PB1 protein, 127V, 550L, and 672L in PA protein, and 64F and 69P in the M protein. These results show that the detected strain had undergone intrasubtype reassortment. Furthermore, it contains changes in the viral proteins that make it more likely to be virulent, raising a question about the tendency of AIV H9N2 to become highly pathogenic in the future for both poultry and humans.

**Supplementary Information:**

The online version contains supplementary material available at 10.1007/s00705-024-06018-2.

## Introduction

In 1966, turkeys in the United States were the source of an avian influenza virus (AIV) H9N2 strain [1], which then split into Eurasian and American lineages [2]. According to Lee and Song [3], the Eurasian group has been further divided into the Korean lineage, the Y280 lineage, and the G1-like lineage, with the G1-like lineage being the most prevalent of these [3, 4].

In 2010, H9N2 G1-like lineages in Egypt were reported [5–7], where the virus subsequently became prevalent in domestic poultry. Based on phylogenetic analysis, Egyptian H9N2 viruses are classified into two genotypes: genotype I, which was found in Egyptian poultry between 2010 and 2013, and genotype II, which emerged in 2014 as a result of reassortment between AIV H9N2 G1 and AIV H9N2 Eurasian strains from wild birds [8, 9].

Influenza A virus is a member of the family Orthomyxoviridae. Its genome consists of eight dsRNA segments that encode at least 10 viral proteins and are arranged in ascending order as follows: polymerase basic protein 2 (PB2), polymerase basic protein 1 (PB1), polymerase acidic protein (PA), hemagglutinin (HA), nucleoprotein (NP), neuraminidase (NA), matrix proteins (M1 and M2), and non-structural proteins (NS1 and NS2 or nuclear export protein [NEP]) [10].

Despite being categorized as low-pathogenicity avian influenza viruses (LPAIs), H9 viruses have been known to exhibit a highly pathogenic phenotype in both laboratory and field settings due to mutations that increase virulence and lethality [11, 12]. Due to their zoonotic potential, H9N2 viruses also represent a threat to human health worldwide, especially since multiple human infections have already been reported [13–16].​ Currently, the capability of H9N2 viruses to contribute their genes to other AIVs that can cross the species barrier to infect humans, such as zoonotic H5N6 [17] and H7N9 [18], is of particular concern worldwide.

Numerous investigations have been carried out to identify viral factors that are linked to increased pathogenicity, virulence, and transmissibility. Specifically, genetic changes associated with airborne transmissibility and adaptation to replication in mammalian hosts are of particular importance [19]. Changes that affect viral entry, viral polymerase activity, and the host response are the main factors determining virulence, and they also affect the efficiency of infection and spread to new hosts [20].

Influenza viruses use both reassortment and individual mutations to adapt to their hosts [21]. Since their first discovery in 1966, H9N2 viruses have undergone evolution and reassortment with other viral subtypes, resulting in a significant increase in their genetic diversity [22]. According to Pusch and Suarez [23], only the G1 and Y280/G9 lineages of H9N2 viruses have been verified to be infectious in humans.​

The current investigation was carried out in order to trace the evolution of Egyptian H9N2 viruses and to determine whether new reassortment events that increase the zoonotic potential and virulence of these viruses are likely to occur.​

## Materials and methods

### Sample collection

One hundred oropharyngeal and cloacal swabs were collected from birds with respiratory manifestations from Egyptian commercial poultry farms (chickens, ducks, and turkeys) with a 15–25% mortality rate during the period from 2020 to 2021. A total of 5 to 10 individual oropharyngeal and/or cloacal swabs collected from each farm were pooled together and treated as one sample. The samples were obtained from 10 Egyptian governorates (Bahira, Dakahlia, Damietta, Giza, Gharbia, Ismailia, Kafr El Sheikh, Menia, Menoufia, and Sharkia). The epidemiological data of the collected samples are provided in Supplementary Table S1.

### Molecular detection and virus isolation

Viral RNA was extracted using a QIAamp Viral RNA Mini Kit (QIAGEN, Hilden, Germany) according to the manufacturer’s instructions in a class 2 biological safety cabinet (SterilGARD, USA). The RNA purity was measured spectrophotometrically using a NanoDrop 2000/2000c instrument (Thermo Fisher Scientific, Waltham, MA, USA). The purity of the extracted viral RNA (A260/A280 ratio) ranged from 1.7 to 2. The extracted RNAs were subjected to quantitative reverse transcription polymerase chain reaction (RT-qPCR) to test for the presence of the influenza A virus matrix (M) gene [24].

Following the standard procedures of the World Organization for Animal Health (OIE) diagnostic handbook, positive samples were inoculated into the allantoic cavities of 9- to 11-day-old specific-pathogen-free embryonated chicken eggs. About 48 hours after inoculation, allantoic fluids were collected, viral RNA was extracted, and RT-PCR was performed for detection of the H5, H9, N8, and N2 genes [25, 26].

## Whole-genome sequencing

Amplicons of each genome segment of an isolate of the H9N2 virus were generated using a SuperScript IV One-Step RT-PCR Kit with Platinum SuperFi DNA Polymerase. The amplicons were sequenced using Ion Torrent next-generation sequencing (NGS) technology (the Ion PGM System with an Ion 316 chip). The reads were analyzed using the Geneious Prime work package (Biomatters, Auckland, New Zealand) as follows: The primer sequences were removed from the raw reads using the “Trim Ends” Geneious Prime plugin. Next, the trimmed reads were mapped using bowtie2, implemented in Geneious Prime, against a reference whole-genome sequence. The resulting sequences were submitted to the GenBank database. The strain was designated A/chicken/Egypt/Menoufia/2021, and the accession numbers are listed in Supplementary Table S2. Whole-genome sequences of H9N2 viruses were downloaded from the NCBI database, and a multiple alignment was made for each genome segment using the Clustal W multiple alignment accessory application in BioEdit software version 7.2.5 (BioEdit Company, Manchester, UK).

### Phylogenetic profiling and molecular characterization

The sequence alignments were used to create a phylogenetic tree by the maximum-likelihood method in MEGA11 software [27], employing the general time-reversible (GTR) nucleotide substitution model. The robustness of the tree branches was estimated using 1000 bootstrap replicates. The antigenic sites and the genetic signature markers associated with virulence, host tropism, enhanced replication, and drug resistance were identified using the aligned amino acid sequences. BioEdit version 7.2.5 was used to compare nucleotide and protein sequences. Potential glycosylation sites were identified using the NetN-Glyc 1.0 server [28]. SWISS-MODEL was used to model the HA protein structures of the Egyptian H9N2 virus and the parental Egyptian AIV H9N2 virus (A/chicken/Egypt/S4456B/2011) [29], and their structures were visualized using PyMOL 1.1 (DeLano Scientific LLC).

## Results

### Sample screening and virus detection

RT-qPCR testing for the avian influenza virus M gene revealed that the virus was present on 23 farms (23%). Three of the positive samples, from farms in Menoufia governorate, contained subtype H9N2. The chickens on these three farms had been vaccinated with a killed H9 vaccine when they were three days old and with an H5 (clade 2.2.1) vaccine when they were eight days old. The mortality rate ranged from 20–22%. Amplification curves and conventional PCR results are shown in Supplementary Figures S1a and b, S2, and S3.

### Phylogenetic profiling and sequence similarity of H9N2 isolates

**Fig. 1:**
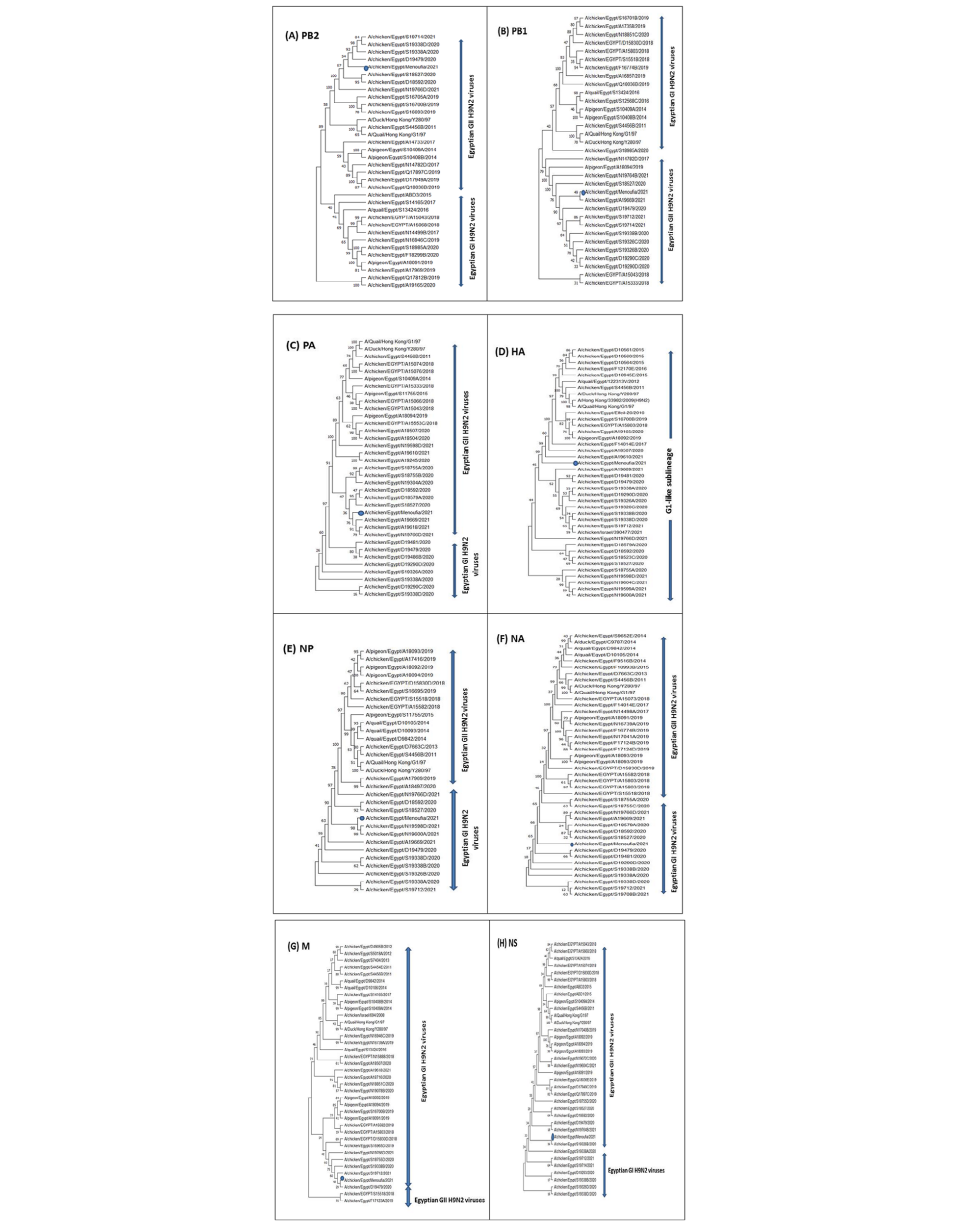
Phylogenetic trees of the eight genome segments (**A**-**H**, respectively), showing the evolutionary relationship of the studied isolate (blue circle) to reference AIVs with sequences in the GenBank database. The trees were constructed using the maximum-likelihood (ML) method in MEGA 11

Phylogenetic analysis based on the surface genes (HA and NA) of the AIV H9N2 virus showed that it belonged to the G1 sublineage of the Eurasian lineage. Our isolate was monophyletic with recent Egyptian AIV H9N2 isolates in the GenBank database, such as A/chicken/Egypt/A19610/2021 and A/chicken/Egypt/N19766D/2021 (Fig. 1), with 99% nucleotide sequence identity in the HA and NA genes (Table 1). However, it was was relatively distant from the parental Egyptian AIV H9N2 virus (A/chicken/Egypt/S4456B/2011) (Fig. 1), with 93% nucleotide sequence identity in the HA gene and 93.5% identity in the NA gene (Table 1). The internal genes (PB2, PB1, PA, NP, M, and NS) of our AIV H9N2 isolate grouped with the G1 sublineage of the Eurasian lineage (Fig. 1), with 99% nucleotide sequence identity to recently published PB1, PB2, M, NP, and NS sequences of Egyptian AIV H9N2 viruses and 97% identity in the PA gene (Table 1). The parental Egyptian AIV H9N2 virus (A/chicken/Egypt/S4456B/2011) was 86%, 89%, 89.55, 94%, 96%, and 89.5% identical in the PB2, PB1, Pa, NP, M, and NS gene, respectively (Table 1). The PB2, PB1, PA, and NS genes showed a close relationship to isolates of Egyptian genotype II (Fig. 1), while the HA, NP, NA, and M genes were more closely related to isolates of Egyptian genotype I (Fig. 1).

**Table 1:** Comparison of the nucleotide sequences of the genome segments of the detected Egyptian H9N2 strain and reference strains

Segment	Reference strain		
	A/chicken/Egypt/S4456B/2011 (First Egyptian parent)	A/pigeon/Egypt/S10408B/2014 (Egyptian genotype II)	A/chicken/Egypt/A19610/2021 (Recently published strain)
PB2	86%	96%	99%
PB1	89%	97%	99%
PA	89.5%	98%	97%
HA	93%	97%	99%
NP	94%	97%	99%
NA	93.5%	96%	99%
M	96%	96%	99%
NS	89.5%	96%	99%

### Molecular characterization

**Fig. 2:**
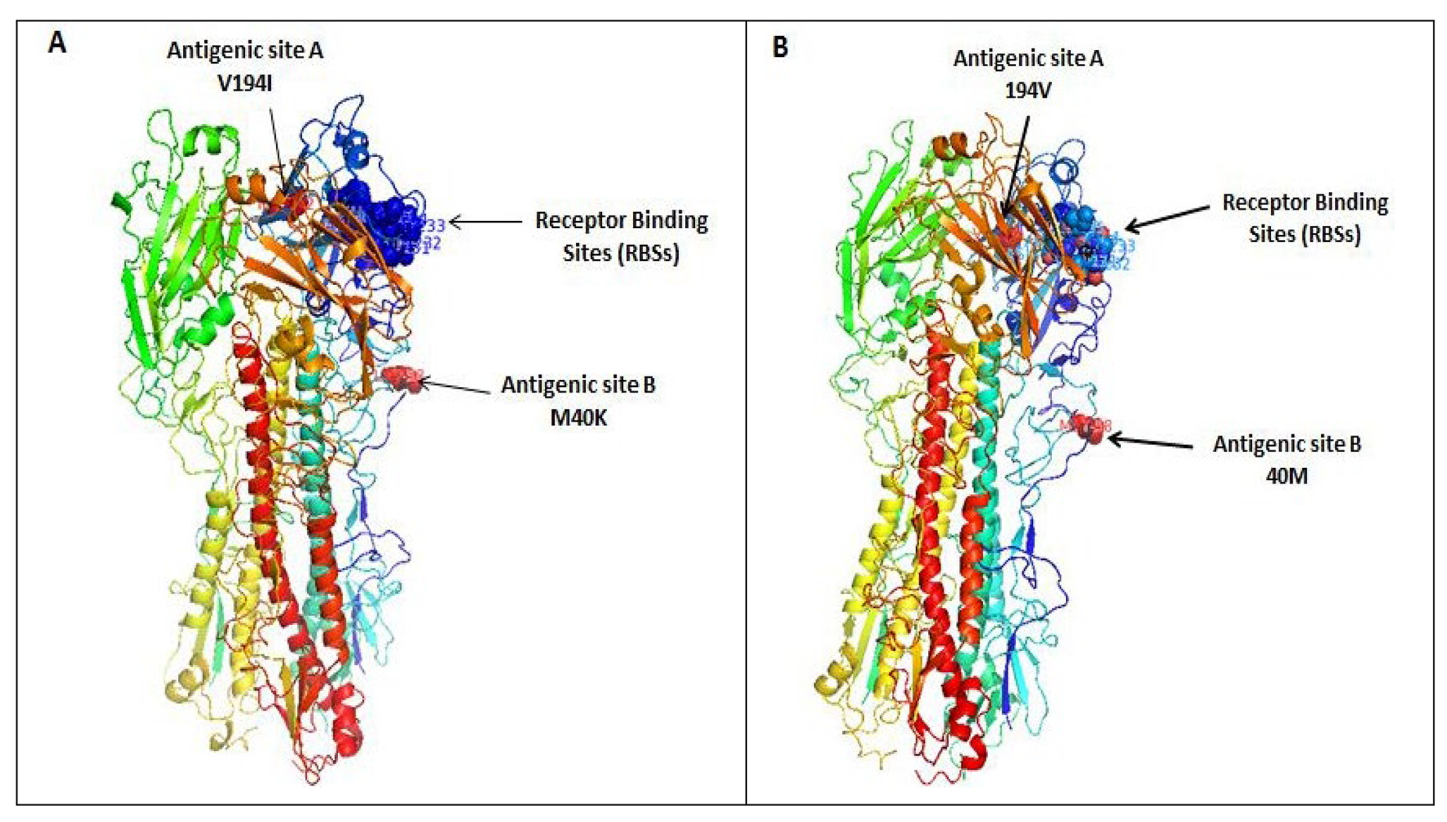
3D structural model of the HA proteins of H9N2 viruses, showing mutations in antigenic sites. (**A**) A/chicken/Egypt/Menoufia/2021 (this study), showing two mutations: V194I and M40K. (**B**) A/chicken/Egypt/S4456B/2011 (Egyptian parental H9N2 virus)

The Egyptian AIV H9N2 isolate from this study has a monobasic motif (PARSSRGLFG) at the cleavage site of the HA protein, which resembles those found in low-pathogenicity AIVs. It also has several amino acid residues in the receptor-binding site (RBS) of the HA that are associated with a preference for binding to human-like α2,6 sialic acid (191H, 232N, 234L, 235I, and 236G) (Fig. 2). In addition, when compared with the parental Egyptian H9N2 virus, the studied strain was found to have gained two substitutions in antigenic sites: V194I in antigenic site A and M40K in antigenic site B (Fig. 2). Moreover, the HA had seven potential N-linked glycosylation sites at positions 29 (NSTE), 82 (NPSC), 105 (NGTC), 141 (NVTY), 298(NSTL), 305(NISK), and 492 (NGTY).

**Fig. 3:**
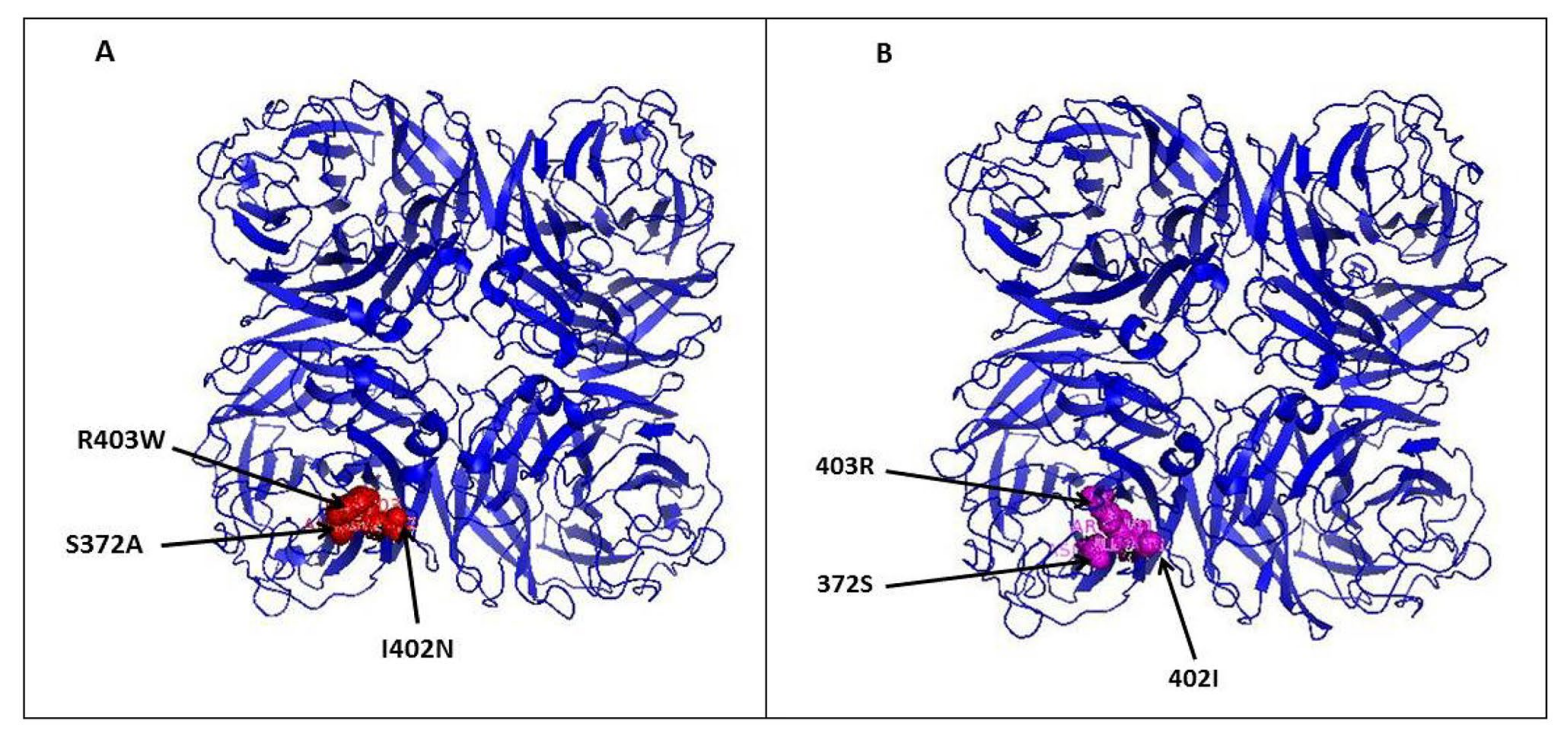
3D structural model of the NA protein showing mutations in haemadsorption sites. (**A**) A/chicken/Egypt/Menoufia/2021 (this study), showing three mutations (R403W, S372A, and I402N). (**B**) The A/Quail/Hong Kong/G1/97 prototype

Genetic analysis of the region encoding the three loops that form the hemadsorption site on the NA gene revealed the presence of three amino acid substitutions: S372A (N2 numbering) in the first loop and I402N and R403W (N2 numbering) in the second loop (Fig. 3) when compared with the A/Quail/Hong Kong/G1/97 prototype (Table 2). In addition, the NA gene has six strong N-linked glycosylation sites at positions 44 (NTST), 61 (NITE), 69 (NGTI), 86 (NWSK), 146 (NGTI), and 234 (NGTC). The polymerase complex (PB2, PB1, and PA) showed the presence of ten markers related to enhanced virulence: V at amino acid position 504 in PB2, 13P, 436Y, 207K, 677T, and 622G in PB1, V at position 127, D at position 383 and L at positions 550 and 672 in PA (Table 3), and three mammalian preference markers: K318R and M64T substitutions in PB2 and D at position 382 in PA.

**Table 2:** Amino acid sequence differences in the NA gene when compared with other selected avian influenza strains

Avian influenza viruses	Hemadsorbing sites			No. of potential glycosylation sites
	1st loop 366–373	2nd loop 399–404	3rd loop 431–433	
A/Quail/Hong_Kong/G1/97	I K K D S R S G	D S D I R	S P Q	6
A/Turkey/Wisconsin/1/1966	I S K D S R S G	D S N N W	S P Q	7
A/Guangdong/MZ058/2016	I K E D S R S G	D S D N W	S P Q	10
**A/chicken/Egypt/Menoufia/2021**	**I K K D S R A G**	**D S N N W**	**P H E**	**6**
A/chicken/Egypt/S4456B/2011	I K K D S R A G	D S D S W	N K Q	6

The M protein was found to have two virulence markers – F at amino acid position 64 and P at position 69 (Table 3) – and three markers associated with mammalian host-specificity: N at position 20, V at position 28, and F at position 55. The NS protein was found to have S at amino acid position 42 and A at position 149, both of which are virulence markers (Table 3). The NP protein was found to have Q at amino acid position 398, which is a unique marker for mammalian host specificity.

**Table 3:** Analysis of molecular markers associated with virulence in the viral proteins of the detected Egyptian H9N2 isolate

Protein	Site	Avirulent	Virulent	A/chicken/Egypt/Menoufia/2021	References
PB2	627 147 250 292 504 588 701 404 591	E M V I I A D F Q	K L G V **V** V N L K	E I V I **V** A D F Q	[60–62]
PB1	317 622	M/V D	I **G**	M **G**	[45, 63, 64]
PA	127 383 224 550 672	I N S I	**V** **D** P **L** **L**	**V** **D** S **L** **L**	[52, 65]
HA	Cleavage site	Monobasic	Multibasic	RSR*GLFG	[59, 65]
NS1	42 92 103 106 149 189	A/P D F M V D/G	**S** E L I **A** N	**S** D F M **A** D	[58, 59, 63, 66, 67]
NS2	31 56	M H/L	I Y	M H	[67]
M2	64 69	P L	S/A/**F** **P**	**F** **P**	[68]
NP	286 437	A T	V M	A T	[69]

## Discussion

Since its identification in Egypt in 2010, the AI H9N2 virus has become prevalent due to its simultaneous circulation alongside clade 2.2.1 H5N1 viruses, which were endemic in the region [30]. In 2017, H5N8 was isolated in Egypt for the first time, and the cocirculation of H9N2 and H5N8 increased the likelihood that reassortment between these two subtypes would result in the appearance of new viruses with pandemic capability. For instance, a recent reassortant (H5N2) between the Egyptian H5N8 and H9N2 viruses has been reported [31, 32]. Furthermore, the zoonotic potential of H9N2 viruses has already been established, with at least 72 verified cases in humans [16].

In the current study, the whole genome sequence of an Egyptian AIV H9N2 virus was determined to evaluate its phylogenetic relationships and to identify molecular genetic markers related to virulence, pathogenicity, and mammalian host preference that were being carried by circulating AIV H9N2 viruses during 2021.

Phylogenetic analysis based on the HA gene showed that the H9N2 isolate A/chicken/Egypt/Menoufia/2021 is closely related to members of a G1-like lineage of H9N2 viruses that were isolated previously in Egypt [9]. Furthermore, a genotype replacement was observed, with phylogenetic analysis revealing that four segments (PB2, PB1, PA, and NS) were associated with genotype II. In contrast, the segments HA, NA, M, and NP were found to be related to those of genotype I viruses. The HA protein in the identified H9N2 strain was predicted to have the monobasic cleavage motif “PARSSRGLF” in the HA1-HA2 connecting peptide. Proteolytic cleavage activation at this site plays a critical role in the viral life cycle [33–35]. Compared with the parental Egyptian AIV H9N2 virus, the isolate from this study has gained two substitutions in antigenic sites: a V194I substitution in antigenic site A and an M40K substitution in antigenic site B. This suggests that antigenic changes have occurred in circulating AIV H9N2 viruses as a result of vaccine failure, illustrating the need for continual updating of commercially used vaccines to match the circulating strains. The H9N2 strain from this study has the 191H and 234L variations, which is associated with a change in the HA preference from avian α-2,3 sialic acid (SA) receptors to human α-2,6 SA receptors, which suggests the potential for binding to human respiratory epithelial cells, as reported by Sorrell *et al.* [36]. Matrosovich *et al.* reported that the 234L variation is characteristic of human pandemic AIV H2 and H3 subtypes. Additionally, the 191H variation is associated with enhanced replication in human respiratory cell cultures and a preference for binding to receptors on human respiratory cells [37–39]. A higher binding affinity of the strain under investigation to human-like receptors is further suggested by the presence of numerous substitutions in the RBS. Genetic examination of the HA sequence revealed that the Egyptian H9N2 strain had seven potential N-linked glycosylation sites, at positions 29 (NSTE), 82 (NPSC), 105 (NGTC), 141 (NVTY), 298 (NSTL), 305 (NISK), and 492 (NGTY). These sites play a crucial role in protein folding, trafficking, pH stability, receptor binding potential, infectivity, and cell-associated host immunological reactions [8, 32, 40]. The N-linked glycosylation site at position 82 has not been seen in recently isolated Egyptian AIV H9N2 viruses. Variations in the glycosylation pattern can have an impact on pathogenicity and the affinity and specificity of receptor binding [9, 41, 42].

Previous studies have shown that the length of the stalk, the positions of N-glycosylation sites, and residues in the enzyme active site are major molecular determinants of the functional activities of NA and that hemadsorption (sialic acid binding) enhances the catalytic efficiency of NA, and therefore, modifications in these locations could potentially affect the specificity of the host receptor and sialic acid binding [42]. In the current study, no stalk deletion was observed in the H9N2 isolate, but three amino acid substitutions were observed in the hemadsorption sites in comparison to the A/Quail/Hong Kong/G1/97 prototype: S372A, I402N, and R403W (N2 numbering). The S372A and R403W substitutions have been shown to enhance the capability of the virus to overcome species barriers and adapt to mammalian hosts. Notably, these substitutions have been observed in the H2N2 and H3N2 subtypes, contributing to pandemics in the human population [42–44]. In addition, the H9N2 strain in this study has six potential N-linked glycosylation sites in the NA gene. This pattern of glycosylation facilitates the cleavage of NA by cellular proteases, which in turn facilitates the spread of infection [9].

The internal proteins (PB2, PB1, PA, NP, M, and NS) of avian influenza virus also influence host tropism and pathogenicity [45, 46], Mutations in the replication complex genes, (PB2, PB1, and PA) have the potential to increase the viral replication rate [47]. In the present study, nine markers related to enhanced polymerase activity and increased virulence were found in components of the polymerase complex: 504V in PB2 [48], 13P, 436Y, 207K, 677T, and 622G in PB1 [49], and 127V, 550L, and 672L in PA [5, 45]. The PA gene plays a major role in the ability of the virus to adapt to new hosts [50, 51]. The PA protein of the studied isolate retained a D residue at position 383, which is present in avian influenza viruses and the G1 prototype strain. This residue may facilitate the crossing of species barriers, as it is linked to increased polymerase activity in avian and mammalian cell lines [52]. The combination of these mutations may enhance polymerase activity. NP plays several roles in the AIV life cycle and its pathogenicity, replication ability, and infectivity in mammals [53]. In this study, the NP protein had the amino acid residue Q at position 398, which is a unique marker of mammalian host preference. A number of variations associated with host tropism and the immune response were found in the M1 and M2 proteins [54, 55]. The M2 protein of our isolate contains markers related to virulence and mammalian host preference: the 64S and 69P variations, which are associated with increased virulence [5], and the 20N, 28V, and 55F variations, which are associated with mammalian host preference and therefore mammalian transmission and human cases [56]. The NS1 protein of our isolate has a PDZ ESEI (227–230) C-terminal motif, which is specific for avian species and is considered a virulence marker [56, 57]. In addition, the NS1 protein has the amino acid residue S at position 42 and A at position 149, both of which have been linked to elevated virulence and more-efficient viral replication in mammalian cells [58, 59].

Our study was limited by the number of sequenced strains and was confined to 10 Egyptian governorates. Thus, it is necessary to conduct additional research on larger numbers of samples to track the evolution of these strains in the field and evaluate their zoonotic potential. Also, an assessment of commercially used vaccines is recommended, and vaccines should be updated periodically. Further research employing animal models is necessary to examine the pathogenicity of the current H9N2 strains in Egypt.

## Conclusion

We determined the whole genome sequence of an AIV H9N2 virus identified in broiler chickens showing respiratory signs in an outbreak with 22% mortality. Phylogenetic analysis revealed that this virus is related to members of the G1 sublineage of the Eurasian group. The studied virus had undergone intra-subtype reassortment, with four segments inherited from Egyptian genotype 1, while the other four segments were inherited from Egyptian genotype 2. This strain showed a number of non-synonymous mutations that are molecular markers of increased virulence, antigenic variability, and affinity for human-like receptors. Interestingly, one substitution was related to H2 and H3 subtypes. The combination of the detected markers indicates the continuous evolution of AI H9N2 in the field and highlights its potential to become highly pathogenic in both poultry and humans.

### Electronic Supplementary Material

Below is the link to the electronic supplementary material

Supplementary Material 1
