# Supplementary material for: Molecular characterization of the whole genome of H9N2 avian influenza virus isolated from Egyptian poultry farms
Source: Arch Virol. 2024 Apr 16;169(5):99. doi: 10.1007/s00705-024-06018-2 (PMC11021324; doi:10.1007/s00705-024-06018-2)
Supplement: Supplementary file 1 — Supplementary Material 1 [file 705_2024_6018_MOESM1_ESM.docx]

**Supplementary data**

**Table (1): Epidemiological data of avian influenza positive samples:**

| No | Date of collection | Age/days | Bird type | Governorate | Vaccination regime | Mortality percent |
| --- | --- | --- | --- | --- | --- | --- |
| 1 | September 2020 | 25 | Chicken (Broilers) | Menoufia | H5(2.2.1) | 22% |
| 2 | October 2020 | 22 | Chicken (Broilers) | Menoufia | H5(2.2.1) | 20% |
| 3 | February 2021 | 24 | Chicken (Broilers) | Menofia | H9(killed) at 3 days of age  H5(2.2.1) at 8 days age | 20% |
| 4 | February 2021 | 25 | Chicken (Broilers) | Menoufia | H5(2.2.1) | 21% |
| 5 | February 2021 | 23 | Chicken (Broilers) | Giza | H5(2.3.2) | 15% |
| 6 | February 2021 | 23 | Chicken (Broilers) | Giza | H5(2.3.2) | 20% |
| 7 | February 2021 | 40 | Duck | Ismailia | H5(2.2.1) | 23% |
| 8 | March 2021 | 23 | Chicken (Broilers) | Damietta | H5(2.2.1) | 23% |
| 9 | March 2021 | 35 | Duck | Ismailia | H5(2.2.1) | 20% |
| 10 | March 2021 | 35 | Duck | Ismailia | H5(2.2.1) | 19% |
| 11 | March 2021 | 40 | Turkey | Giza | H5(2.2.1) | 25% |
| 12 | March 2021 | 22 | Chicken (Broilers) | Menoufia | H9(killed) at 3 days of age  H5 at 8 days age | 22% |
| 13 | March 2021 | 25 | Chicken (Broilers) | Menoufia | H9(killed) at 3 days of age  H5 at 8 days age | 20% |
| 14 | March 2021 | 25 | Chicken (Broilers) | Gharbia | H5(2.2.1) | 18% |
| 15 | March 2021 | 25 | Chicken (Broilers) | Dakahlia | H5(2.2.1) | 22% |
| 16 | April 2021 | 25 | Chicken (Broilers) | Beheira | H5(2.2.1) | 18% |
| 17 | April 2021 | 20 | Chicken (Broilers) | Beheira | H5(2.2.1) | 15% |
| 18 | May 2021 | 22 | Chicken (Broilers) | Damietta | H5(2.2.1) | 20% |
| 19 | May 2021 | 35 | Duck | Menia | H5(2.2.1) | 22% |
| 20 | May 2021 | 40 | Duck | Gharbia | H5(2.2.1) | 24% |
| 21 | May 2021 | 40 | Duck | Sharkia | H5(2.2.1) | 19% |
| 22 | June 2021 | 45 | Turkey | Sharkia | H5(2.2.1) | 20% |
| 23 | July 2021 | 45 | Turkey | Kafr El Sheikh | H5(2.2.1) | 18% |

**Table (2): Accession numbers of the 8 segments of the detected strain A/chicken/Egypt/Menoufia/2021(H9N2):**

| Accession number | Name |
| --- | --- |
| OP115742 | Influenza A virus (A/chicken/Egypt/Menoufia/2021(H9N2)) segment 1 polymerase PB2 (PB2) gene, complete cds |
| OP115741 | Influenza A virus (A/chicken/Egypt/Menoufia/2021(H9N2)) segment 2 polymerase PB1 (PB1) and PB1-F2 protein (PB1-F2) genes, complete cds |
| OP115743 | Influenza A virus (A/chicken/Egypt/Menoufia/2021(H9N2)) segment 3 polymerase PA (PA) and PA-X protein (PA-X) genes, complete cds |
| OP115744 | Influenza A virus (A/chicken/Egypt/Menoufia/2021(H9N2)) segment 4 hemagglutinin (HA) gene, complete cds |
| OP115747 | Influenza A virus (A/chicken/Egypt/Menoufia/2021(H9N2)) segment 5 nucleocapsid protein (NP) gene, complete cds |
| OP115745 | Influenza A virus (A/chicken/Egypt/Menoufia/2021(H9N2)) segment 6 neuraminidase (NA) gene, complete cds |
| OP115746 | Influenza A virus (A/chicken/Egypt/Menoufia/2021(H9N2)) segment 7 matrix protein 2 (M2) and matrix protein 1 (M1) genes, complete cds |
| OP115748 | Influenza A virus (A/chicken/Egypt/Menoufia/2021(H9N2)) segment 8 nuclear export protein (NEP) and nonstructural protein 1 (NS1) genes, complete cds |


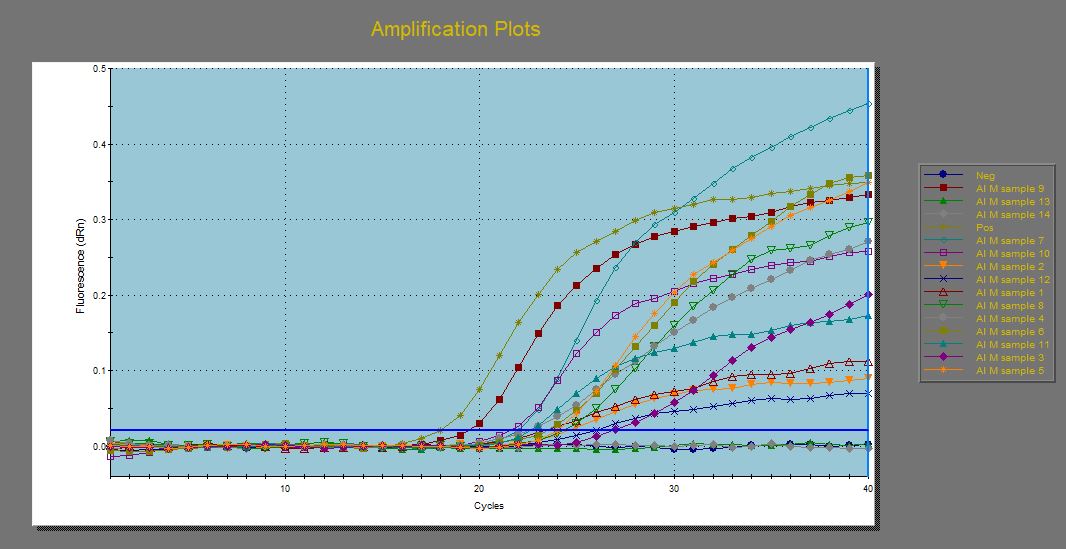


**Fig. (1a):** Amplification curves of examined samples including negative and positive controls.


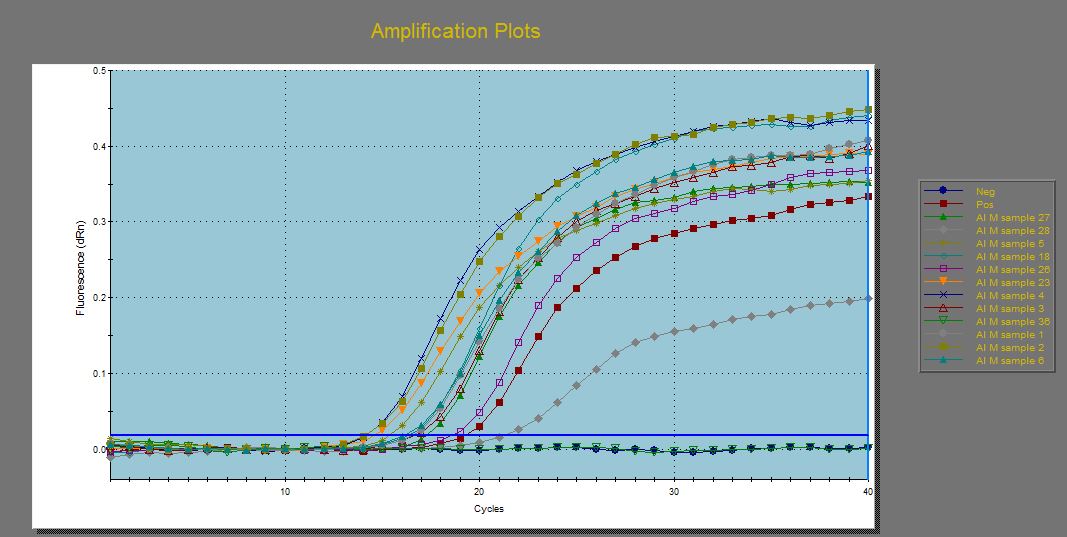


**Fig. (1b)**: Amplification curves of examined samples including negative and positive controls.


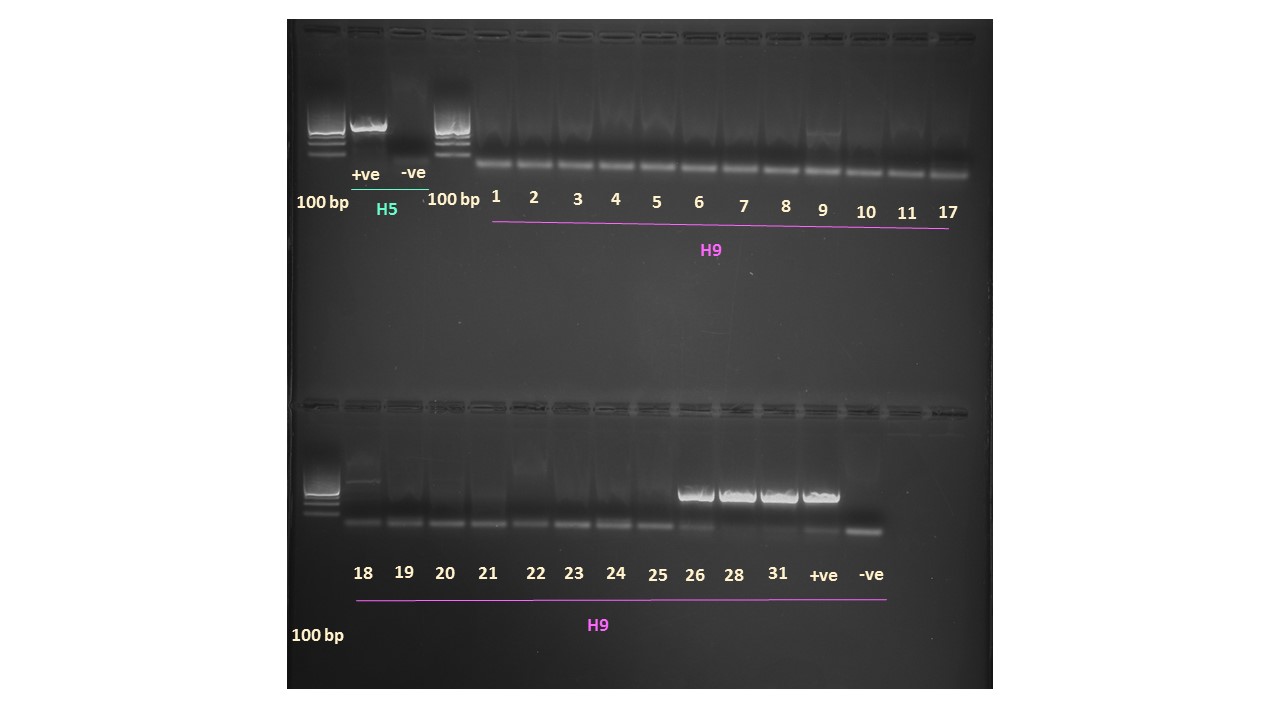


**Fig. (2)**: Gel electrophoresis of amplified HA gene. H9 Positive samples have 488 bp band.


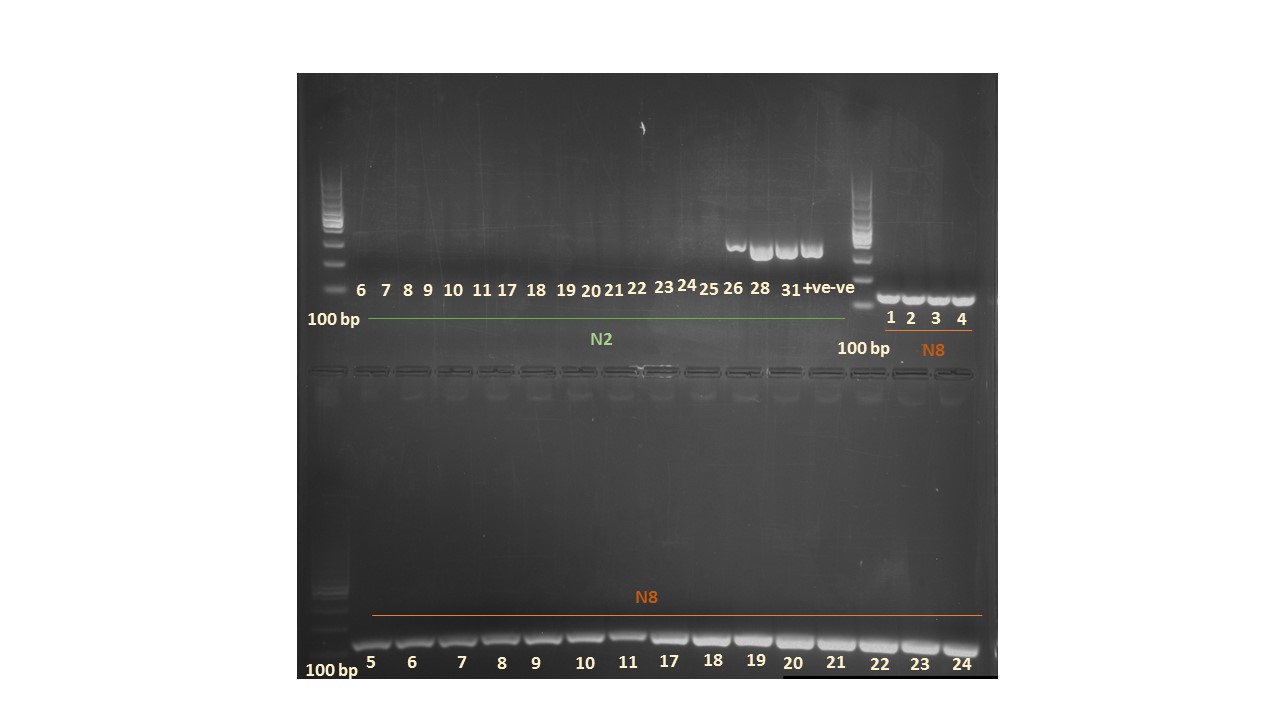


**Fig. (3)**: Gel electrophoresis of amplified NA.N2 positive samples have 278 bp band.
